# Efficacy and Safety of COVID-19 Vaccination in Older Adults: A Systematic Review and Meta-Analysis

**DOI:** 10.3390/vaccines11010033

**Published:** 2022-12-23

**Authors:** Lei Zhang, Lihong Jiang, Tian Tian, Wenjing Li, Yonghui Pan, Yongchen Wang

**Affiliations:** 1 Department of General Medicine, The Second Affiliated Hospital of Harbin Medical University, Harbin 150086, China; 2 Department of Epidemiology, School of Public Health, Harbin Medical University, Harbin 150086, China; 3 Fourth Department of Neurology, The First Affiliated Hospital of Harbin Medical University, Harbin 150001, China

**Keywords:** COVID-19, vaccines, elderly, effectiveness, security

## Abstract

Objective: To analyze the differences in efficacy and safety of different types of novel coronavirus pneumonia (COVID-19) vaccines in different age groups (young adults and elderly). Methods: Randomized controlled trials (RCTs) on COVID-19 vaccine in PubMed, Embase, Web of Science, and Cochrane library were searched by computer, and eight eligible studies were analyzed. Meta-analysis was performed using Stata 16.0 and RevMan5.4 software. Results: The mean geometric titer (GMT) of the virus in the elderly was significantly higher than that in the placebo group (SMD = 0.91, 95% CI (0.68, 1.15), *p* < 0.01), presenting no obvious difference compared with the young adults (SMD = 0.19, 95% CI (0.38, 0.01), *p* = 0.06). Meanwhile, the effect of multiple vaccinations was better than that of single vaccination (SMD = 0.83, 95% CI (0.33, 1.34), *p* < 0.01). However, the number of adverse events (AEs) in the elderly was lower than that in the young adults (OR = 0.35, 95% CI (0.29, 0.42), *p* < 0.01). Conclusions: The immunization effect of COVID-19 vaccine in the elderly is obvious, especially after multiple vaccinations, and the incidence of AEs in the elderly is low, which proves that the vaccination of the elderly is safe and effective.

## 1. Introduction

COVID-19 is an acute respiratory infectious disease caused by severe acute respiratory syndrome coronavirus 2 (SARS-CoV-2), which has spread widely around the world, caused devastating medical, economic, and social consequences [1,2,3]. As of 1 November 2022, more than 627.56 million people have been diagnosed with COVID-19 globally, with more than 6.58 million deaths. Common manifestations of COVID-19 include cough, fever, sore throat, reduced sense of smell and taste, and even difficulty breathing [4]. COVID-19 spreads more easily from person to person by droplets and has a higher infection rate but a lower death rate than SARS, MERS, and influenza. Given these characteristics, it has spread extremely quickly from its source to other regions and countries, becoming a serious public health event.

People are generally susceptible to COVID-19, the immune function of the elderly is weakened, and most of them are complicated with chronic underlying diseases. After infection, they often progress rapidly and most of them are severe cases. The risk of hospitalization and death from COVID-19 is significantly higher among the elderly and those with chronic underlying conditions. Epidemiological data clearly show that the morbidity and mortality of elderly patients are significantly higher than those of young patients [5,6,7,8]. The main risk factors for death from COVID-19 include age, race, frailty, and comorbidities, such as diabetes, hypertension, cerebrovascular disease, cardiovascular disease, chronic obstructive pulmonary disease, chronic kidney disease, and other factors, such as obesity and smoking [9,10,11,12,13,14,15,16]. Mortality rates also varied by ethnicity, with higher infection and death rates among African Americans or blacks, while African mortality remained relatively low during the early stages of the pandemic [17]. The COVID-19 pandemic has revealed many deficiencies in the understanding of the mechanisms underlying aging-related disease susceptibility and maladaptation. Therefore, protecting the elderly population is particularly critical in the prevention and control of the novel coronavirus pneumonia epidemic. Infection control measures, such as wearing masks, suspending public gatherings, closing schools, and restricting travel, are an effective means of prevention despite their severe impact on daily life and the global economy. At first, dexamethasone was given to patients in hospitals. Now, with the introduction of new effective antiviral drugs, the situation has begun to change, and vaccination is the most effective prevention and treatment measure. Therefore, the development of COVID-19 vaccine has become an urgent problem for all countries in the world [18]. Existing COVID-19 vaccines include inactivated, live attenuated, recombinant protein, carrier, and nucleic acid vaccines. More than 90 vaccines against SARS-CoV-2 are being evaluated in various clinical trials, and several have been approved for mass vaccination, such as BNT162b1 (PCI-Biontech), CoronaVac (Sinovac), Ad26.COV2-s (Johnson & Johnson), KCONVAC (Kangtai) Biological), ChAdOx1-s (Oxford-AstraZeneca), NVX-CoV2373 (Novavax), BBIBP-CorV (Sinopharm), V-01 (Livzon Pharmaceutical Group Inc.) etc. [19,20,21]. BBIBP-CorV, CoronaVac, and KCONVAC are the first inactivated vaccines developed after the COVID-19 outbreak. Such vaccines use the most traditional platforms, using physical or chemical methods to inactivate the virus but retain its immunogenicity. When inactivated viruses enter the body, they cause an immune response but are not pathogenic. This vaccine technology is considered to be the safest and easy to develop, so it is often used to contain emerging infectious diseases [22,23]. Viral vector vaccines include Ad26.COV2-s and ChAdOx1-s are vectors derived from the recombinant spike genes of adenovirus and SARS-COV-2, which stimulate cellular and humoral immunity to produce a more durable and effective immune response than inactivated vaccines and have been widely used in various countries and regions [24,25]. BNT162b1 was developed using a platform based on the coding of viral protein MRnas, encoded fragments of which will be injected into the human body, translated into antigenic proteins in human cells, and induce immune responses in the human system. mRNA vaccines are being developed faster than vaccines based on other platforms. mRNA vaccines have been shown to provide adequate protection, but the technology to develop them is new and has not been used to mass-produce vaccines or prevent infectious diseases. Therefore, long-term monitoring of the safety and effectiveness of these vaccines is needed. In addition, the storage conditions required for these vaccines make them difficult to transport due to the instability of mRNA structure [26]. Recombinant protein vaccines include NVX-CoV2373 and V-01. The basic technological principle is to identify specific proteins of the pathogen with immunogenicity, integrate the specific protein genes of the pathogen into yeast and other microorganisms through genetic engineering method, and express the specific proteins of the pathogen in large quantities in vitro, and prepare the vaccine after purification. With the preparation of pathogen-specific proteins in vitro, human body can be stimulated to produce antibodies [27,28]. Previous studies have shown that seroconversion occurs in the vast majority of vaccinated individuals, but GMT is significantly lower in people aged 60 years and older [29,30]. In addition, the immunogenicity of inactivated vaccines such as influenza has been shown to be more limited in the elderly [31]. There was little change in the number of antibodies of any isotype that people produced as they grew older. However, the quality of the antibody response changes, with older adults producing fewer antibodies specific against the activated pathogen or vaccine [32]. 

This study evaluated the safety and effectiveness of the current COVID-19 vaccine in the elderly by means of evidence-based meta-analysis, taking the efficacy and adverse reactions of the vaccine as indicators, in order to provide evidence from another perspective for the development of prevention and control strategies for COVID-19, and to develop effective public health strategies during the pandemic.

## 2. Method

This meta-analysis follows the PRISMA statement and has been registered on the PROSPERO platform (CRD42022343986) [33].

### 2.1. Search Strategy

PubMed, Embase, Web of Science, and Cochrane library databases were searched by computer to collect relevant literatures. The search is conducted by combining subject words and free words. Key search terms include: “COVID-19”, “older adults” and “vaccines” and “randomized controlled trials”. The search deadline is 1 May 2022. Detailed retrieval strategies in the case of PubMed are presented in the supplementary document.

### 2.2. Eligibility Criteria

Randomized controlled trials and observational studies of efficacy of COVID-19 vaccines are qualified. The primary endpoint of the effectiveness index is GMT. The primary endpoint of the safety indicator is the number of AEs occurrences. We included only fully vaccinated and unvaccinated populations and compared the efficacy and safety of the vaccine in such populations. We excluded some of the vaccinated people. The inclusion criteria of this meta-analysis were as follows: (1) the elderly group (age ≥ 60 years) was included; (2) to report data on the effectiveness and safety of COVID-19 vaccines; (3) randomized controlled trials. We excluded studies that: (1) conference abstracts, letters, and comments; (2) retrospective study; (3) studies in which basic data cannot be retrieved; (4) the same patients were recruited in different studies. 

### 2.3. Study Selection

First, one author (L.Z.) was selected by reading the title and abstract of the studies, and then two authors (L.Z., W.L.) reviewed the full text of the selected studies and cross-checked them. Any disagreement was resolved through discussion with the third author (Y.W.). Reasons for inclusion or exclusion were recorded. 

### 2.4. Data Extraction

Data extraction contents include: (1) basic information included in the study; (2) basic characteristics of the research object; (3) the time, dosage form, and dosage of vaccination; (4) Results: effectiveness and adverse reactions. All kinds of data should be extracted directly from the original text as far as possible. If there are any missing data, SD, CI, and other indicators are used for estimation according to Cochrane Manual of Systematic Review. If SD, CI, and other indicators are missing, SD of other studies are used for average value. If important specific data are missing, we contact the first author of the original article to obtain unpublished data and other details.

### 2.5. Comprehensive Data Analysis

The meta-analysis was performed using STATA 16.0.

The random-effects model was used for analysis. The preliminary analysis reported the effectiveness of vaccination against COVID-19 in the elderly, and the primary outcome measure was 95% CI of GMT, the end point of vaccine effectiveness. Study heterogeneity was assessed by I^2^, with I^2^ ≥ 50% as significant heterogeneity [34]. 

In addition, we investigated the safety of COVID-19 vaccination for older adults. To achieve this, the research team conducted the analysis with free access to the data or contacted the corresponding authors of the included articles. Because the data from the multivariate analysis already conducted in the original study were best suited to identify the factors associated with the outcome (i.e., adverse reactions to COVID-19 vaccination), we used the most adjusted model for every study with these data and extracted the ORs for the common factors in the study. In this way, we analyzed vaccine adverse reactions. *p* < 0.05 was considered statistically significant. 

## 3. Results

### 3.1. Search Results

**Figure 1:**
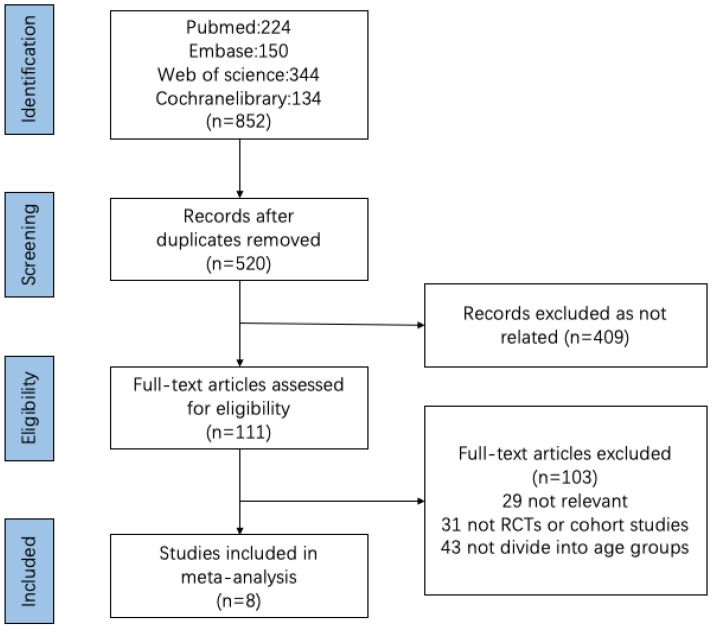
Flowchart of studies evaluating qualified research through selection process.

We obtained 852 references from the database, including 332 duplicates. A total of 409 articles were deleted after reading the title and abstract. Of the remaining 111 articles, 103 were removed after a detailed reading and evaluation of the full text. In the end, eight studies were included in the vaccine efficacy and safety analysis. The selection flow chart is shown in Figure 1. 

### 3.2. Characteristics of the Study

The included vaccine efficacy or safety analysis included a total of 4543 people from eight studies, including 2441 under 60 years of age and 2102 over 60 years of age. In addition, Maheshi et al. included an additional group of 160 people aged 56–69 years of age. All the included studies were randomized controlled trials. Five of the studies were conducted in China, one in Belgium and the United States, one in the United Kingdom, and one in the United States and Australia. The included applications were inactivated vaccine (CoronaVac, BBIBP-CorV, KCONVAC), recombinant protein vaccine (NVX-CoV2373, V-01), viral vector vaccine (Ad26.COV2, ChAdOx1), and RNA vaccine (BNT162b1). All studies were evaluated for quality using the modified Jadad scale, with scores between 6 and 7, indicating that the included studies were of high quality. The detailed information of each included article is shown in Table 1 [22,23,24,25,27,28,35,36]. 

### 3.3. Effectiveness of COVID-19 Vaccination for Older Adults

**Figure 2:**
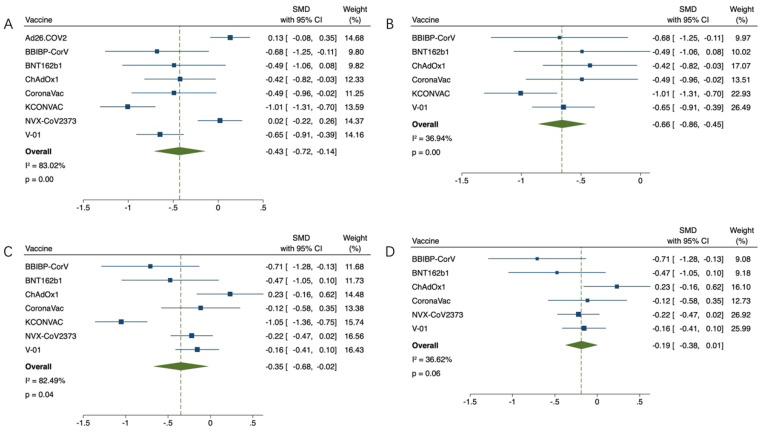
(**A**,**B**) Meta-analysis of GMTS between the experimental group (older adults) and the control group (younger adults) after the first vaccination, and results after reducing heterogeneity. (**C**,**D**) Meta-analysis of the GMTS of the experimental group (older adults) and the control group (young adults) after the last vaccination, and results after reducing heterogeneity. Blue squares represent effect sizes for a single study, and green rhombus represent pooled results for all studies.

**Figure 3:**
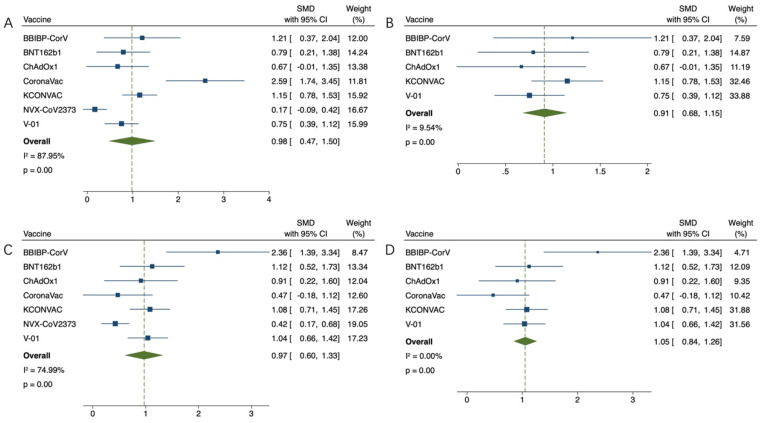
(**A**,**B**) Meta-analysis of the GMT between the experimental group (older adults) and the control group (placebo) after the first vaccination, and results after reducing heterogeneity. (**C**,**D**) Meta-analysis of the GMTS of the experimental group (elderly) and the control group (placebo) after the last vaccination, and results after reducing heterogeneity. Blue squares represent effect sizes for a single study, and green rhombus represent pooled results for all studies.

**Figure 4:**
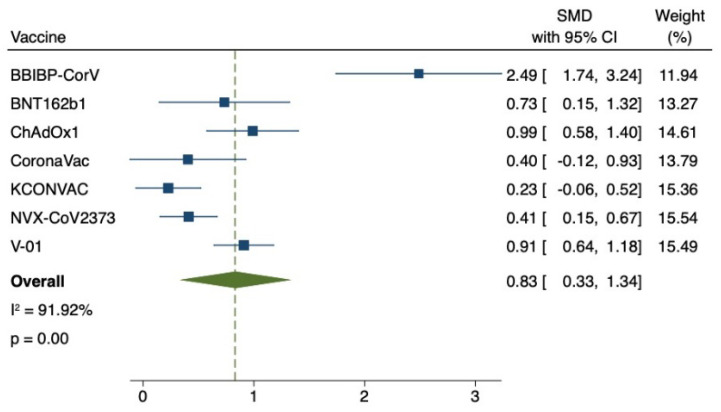
Meta-analysis of GMTS after the last vaccination versus the first vaccination in older adults. Blue squares represent effect sizes for a single study, and green rhombus represent pooled results for all studies.

The effect index was the GMT value at 21 or 28 days after the first inoculation and at 14, 21 or 28 days after the last inoculation. GMT value was positively correlated with immunogenicity. The GMT value of the virus was lower in the older group than in the younger group (SMD = −0.43, 95% CI (−0.72, −0.14), *p* < 0.01) (Figure 2A). Due to high heterogeneity (83.02%), the sensitivity analysis found that the works of J. Sadoff et al. on Ad26.COV2.S and Neil Formica et al. on NVX-CoV2373 had the lowest confidence interval overlap rate. Heterogeneity (36.94%) returned to normal after excluding (SMD = 0.66, 95% CI (0.86, 0.45), *p* < 0.01) (Figure 2B). Subsequently, GMT values of each group after the last vaccination were compared. In the study of Ad26.COV2.S vaccine by J. Sadoff et al., elderly people did not receive the second injection. The results of other studies were compared as follows: compared with the youth group, the GMT value of the virus in the elderly group was lower (SMD = −0.35, 95% CI (−0.68, −0.02), *p* < 0.05) (Figure 2C), due to high heterogeneity (82.49%), sensitivity analysis found that Jiankai Liu et al.’s work on KCONVAC vaccine had the lowest confidence interval overlap rate. Heterogeneity (36.62%) returned to normal after excluding (SMD = 0.19, 95% CI (0.38, 0.01), *p* = 0.06) (Figure 2D). The final results indicated that there was no significant difference in immunogenicity between the old group and the young group, and there was a good efficacy index. This study then compared the elderly who received the vaccine with the elderly who received the placebo. After adjusting the upper and lower GMT values of the placebo group, it was found that the GMT values of the elderly vaccinated group were much higher than those of the placebo group in the first vaccination comparison (SMD = 0.98, 95% CI (0.47, 1.50), *p* < 0.01) (Figure 3A). Due to high heterogeneity (87.95%), sensitivity analysis found that Gang Zeng et al.’s study on CoronaVac vaccine and Neil Formica et al.’s study on NVX-CoV2373 vaccine had the lowest confidence interval overlap. After removal, the heterogeneity (9.54%) returned to normal (SMD = 0.91, 95% CI (0.68, 1.15), *p* < 0.01) (Figure 3B). The GMT value of the vaccine group was significantly higher than that of the placebo group (SMD = 0.97, 95% CI (0.60, 1.33), *p* < 0.01) (Figure 3C). Due to high heterogeneity (74.99%), the sensitivity analysis found that Neil Formica et al.’s NVX-CoV2373 vaccine had the lowest confidence interval overlap and, after removal, the heterogeneity (0.00%) returned to normal (SMD = 1.05, 95% CI (0.84, 1.26), *p* < 0.01) (Figure 3D). The above results suggest that the immune effect of the elderly vaccinated with COVID-19 vaccine is far better than that of the placebo control group, with more advantages in the efficacy indicators. Among them, J. Sadoff et al.’s article showed no direct data on the placebo indicators, but the conclusions of the literature also supported the conclusions of the above meta-analysis. Finally, the GMT value of the virus after the last injection of the vaccine was compared with that after the first injection of the vaccine. The GMT value of the virus after the last injection of the vaccine was higher than that after the first injection (SMD = 0.83, 95% CI (0.33, 1.34), *p* < 0.01) (Figure 4). It is proved that multiple injections of the vaccine in the elderly can improve the immunogenicity and enhance the effect of the vaccine. 

### 3.4. Safety of COVID-19 Vaccination for Older Adults

**Figure 5:**
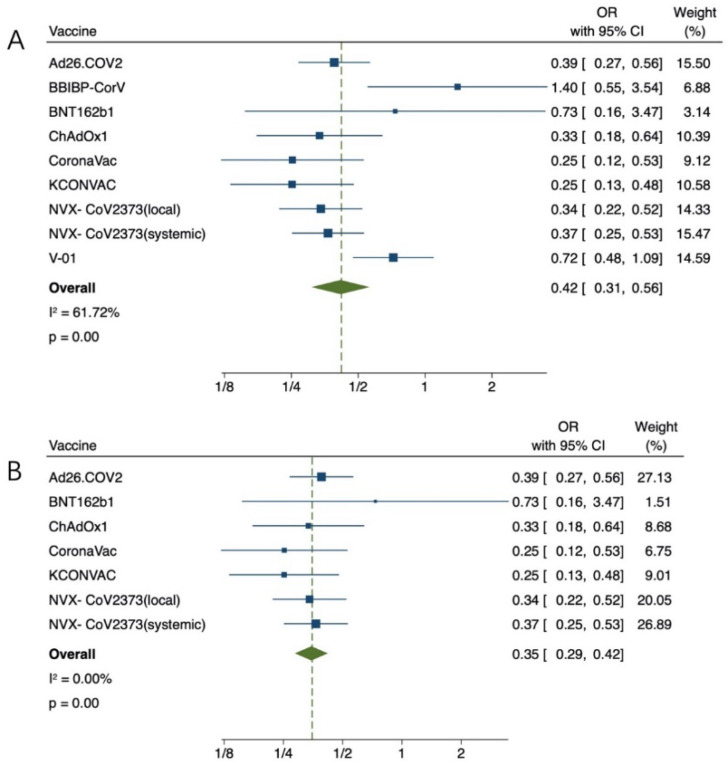
(**A**) Meta-analysis of any AEs between the experimental group (older adults) vs control group (young adults). (**B**) After removing the heterogeneity, Meta-analysis of any AEs between the experimental group (older adults) vs control group (young adults). Blue squares represent effect sizes for a single study, and green rhombus represent pooled results for all studies.

Common local AEs include pain, itching, redness, and sclerosis at the injection site. Common systemic AEs include fever, chills, nausea, headache, diarrhea, and joint pain. We found that young adults had a higher incidence of AEs than older adults (OR = 0.42, 95% CI (0.31, 0.56), *p* < 0.01) (Figure 5A). Due to high heterogeneity (61.72%), sensitivity analysis found that Shengli Xia et al.’s work on BBIBP-CorV vaccine and Ya-Jun Shu et al.’s work on V-01 vaccine had the lowest overlap rate of confidence intervals. After removal, the heterogeneity (0.00%) returned to normal (OR = 0.35, 95% CI (0.29, 0.42), *p* < 0.01) (Figure 5B). These results suggest that COVID-19 vaccine has more advantages in regard to the safety index of the elderly. 

### 3.5. Risk of Bias

**Figure 6:**
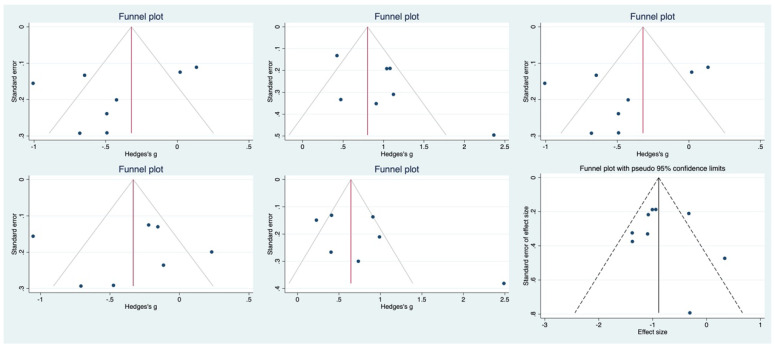
Funnel plot of each comparative analysis. Blue circles represent included studies, solid lines represent pooled effect sizes, dashed lines represent confidence intervals (95%).

**Figure 7:**
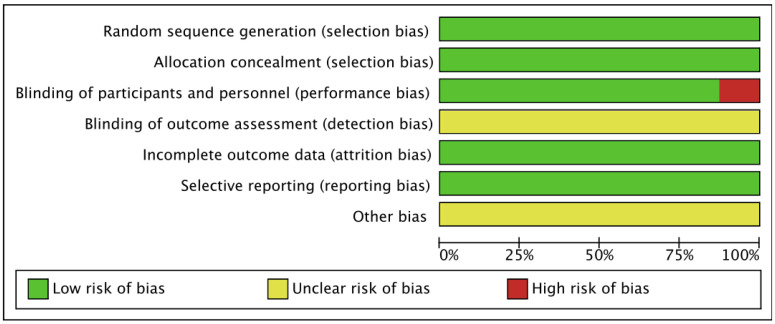
Risk of bias.

The funnel plot shows some asymmetry. Subsequently, the Egger regression test was conducted to compare the effectiveness of COVID-19 vaccine between the elderly and the young, the effectiveness of the elderly and the placebo, and the safety index, and the result was not significant (*p* = 0.247; *p* = 0.114; *p* = 0.247; *p* = 0.862; *p* = 0.197; *p* = 0.701), indicating that publication bias is less likely (Figure 6). The methodological quality of the research was evaluated in a variety of ways using RevMan5.4 software (Figure 7).

## 4. Discussion

The COVID-19 epidemic remains prominent around the world. Due to the introduction of new infectious sources, COVID-19 may break out again at any time [37]. Therefore, we must vaccinate more to immunize more people [38]. Related works included in this study considered BNT162b1, CoronaVac, Ad26.COV2-s, KCONVAC, ChAdOx1, NVX-CoV2373, BBIBP-CorV, and V-01, respectively. The randomized controlled trial of the vaccine described above was a very comprehensive dose study. 

Meta-analysis of efficacy indicators showed that GMT in the elderly group was lower than that in the young group, but it was much higher than that in the placebo control group, suggesting that the effectiveness of vaccination in the elderly group was very significant. In addition, all types of COVID-19 vaccines have shown good efficacy. At the same time, the study also found that older adults who received multiple doses of the vaccine were more effective than those who received a single dose. Different types of vaccines induce antibodies in different ways, leading to differences in vaccine efficacy. Inactivated viral vaccines, such as CoronaVac, BBIBP-CorV, and KCONVAC, showed stable antibody expression, but failed to induce immune memory with a certain probability [39]. mRNA vaccines, such as BNT162b1, as a recently emerging type, encode mRNA with prefixed spike (S) to generate target protein and induce immune response. mRNA vaccines have the highest risk of adverse events, but they are powerful, fast to develop, and cheap to produce. 

Meta-analysis of safety indicators showed that the incidence of adverse reactions in the elderly was lower than that in the young, suggesting that the vaccine had more advantages for the elderly in terms of safety indicators. Any vaccine can cause temporary side effects due to activation of the immune response and tissue damage at the injection site. The most common local and systemic adverse reactions were grade 1 severity, followed by grades 2 and 3. Injection site pain was the most common adverse event associated with any type of COVID-19 vaccine, occurring in 20.29% of participants in the older group and 38.61% in the younger group. The next most common adverse reactions were fatigue and headache, which occurred in 16.28% and 11.85% of the elderly group and 29.30% and 25.87% of the young group, respectively. At the same time, the reason for the low GMT value of the elderly is immune senescence. The changes of immune organs in the elderly are most obvious in the thymus, which has weak functional activity and reduced immune reactive substances, but this also leads to a lower incidence of adverse reactions in the elderly [40,41]. 

After reviewing other literature, we found some reports of adverse reactions related to mRNA vaccines and viral vector vaccines (such as BNT162b2, Ad26.COV2-s and ChAdOx1). Cases of immunothrombotic thrombocytopenia (VITT), pulmonary embolism (PE), disseminated intravascular coagulation (DIC), and cerebral venous sinus thrombosis (CVST) were reported. VITT presents with thromboembolic symptoms, especially signs of thrombocytopenia, cerebral thrombosis, or arterial thrombosis in the abdomen and elsewhere, such as easy bruising, bleeding, severe headache, abdominal pain, numbness, and cold limbs, especially when onset occurs 4–28 days after vaccination. However, to date, the amount of data on such adverse effects is too small to provide clear evidence of causation. At the same time, Johnson & Johnson’s recombinant adenosis vector vaccine was also suspended by FDA due to rare blood-related problems in some people, namely thrombocytopenia syndrome thrombosis, resulting in cerebral venous sinus thrombosis (CVST) [42,43,44,45]. Overall, these life-threatening serious adverse events are rare, so ongoing global vaccination programmes should still be supported. 

More than half of the data in this study were from China, including inactivated vaccines, RNA vaccines, and recombinant protein vaccines (BBIBP-CorV, CoronaVac, KCONVAC, BNT162b1, V-01). The data of the vaccine injected in China showed that the GMT value of the elderly group was lower than that of the young group, but much higher than that of the placebo control group, indicating that the effectiveness of vaccination in the elderly in China was very significant. At the same time, the number of adverse reactions in the elderly was less than that in the young group, indicating that the safety of the above types of vaccines is very good, suggesting that the injection of COVID-19 vaccines has its excellent effect regardless of the country or region. 

The effectiveness of vaccination programmes is decisive for the prevention of COVID-19, especially among those who are likely to be more affected [46,47]. In conclusion, we encourage the elderly and young to get vaccinated against COVID-19 as soon as possible. Previous studies have also confirmed the importance of vaccinating older people against COVID-19. Di Tian et al. demonstrated that existing vaccines cannot provide complete protection against COVID-19, so the vaccinated population still needs to take preventive measures, such as wearing masks, but vaccination can effectively reduce the rate of severe illness, hospitalization, and mortality [48]. The study of Manish et al. proved that unless the vaccine acted directly on the elderly, the total mortality rate would not be significantly reduced simply by vaccinating children and young people and effectively preventing transmission [7]. Giuliana et al. demonstrated that the elderly receive a third/booster dose of the vaccine for increased immune response and possible protection [49]. At the societal level, mathematical analysis by Goldstein et al. shows that for COVID-19, vaccinating the oldest first saves the most lives and effectively prolonging survival [50]. Therefore, those who have not completed the vaccination program should do so as soon as possible, and the coverage of those vaccinated should be expanded, so as to build a solid defense line to control the epidemic. 

Although this study confirmed the safety and effectiveness of vaccination for the elderly, the strategy of vaccination for the young cannot be simply translated to the elderly population, and how to further improve the efficacy and safety of vaccination for the elderly needs to be concerned. It is recommended that the elderly should be vaccinated with adjuvants to enhance the humoral response and increase the antibody titer and serum conversion rate in the elderly [51]. Without affecting safety, high-dose vaccination can enhance the humoral response of elderly patients and improve the efficacy [52]. In terms of improving safety, we can establish and improve the follow-up system, perform thorough short- and long-term follow-up, strengthen the safety assessment of vaccines, and conduct appropriate monitoring and post-marketing safety studies [53]. In addition, the study of Ucciferri C et al. confirmed that the use of pidotimod reduced adverse events related to BNT162b2 vaccination and improved tolerability without interfering with antibody production in subjects [54]. Regarding mechanisms for developing new vaccines for the elderly, the continued development of adjuvants and vaccination techniques to target some of the known deficits in immune responses in older adults is needed to achieve greater efficacy in this population.

Most of the previous single randomized controlled studies on COVID-19 included older and younger adults, but no systematic meta-analysis of these studies has been conducted. Therefore, by analyzing the differences between the efficacy and safety indicators of the elderly after vaccination and those of the young people and those of the placebo control group, this study suggests that the vaccination of the elderly is safe and effective, and further confirms the correctness of China’s vaccine-related decision-making in the prevention and control of COVID-19. The advantages of this study are that the included articles are of high quality and the design of the RCT is reasonable, which can solve some existing questions about vaccine hesitancy in the elderly. This definitely helps to address concerns about vaccine hesitancy, especially among the elderly. However, there are still some limitations to this study. First, due to the short development time of COVID-19, vaccine research time is insufficient, RCT sample size is small, vaccine types are few, and the follow-up time is short. Second, in the included studies, the work of Neil Formica et al. [28] was single-blind rather than double-blind, which may be the cause of selection bias. Third, the meta-analysis of GMT showed great heterogeneity, which may be related to the different effects achieved by different types of vaccines. Therefore, this conclusion needs to be further confirmed by more high-quality, multi-center, large-sample studies.

## 5. Conclusions

There is no significant difference in the immune effect between the elderly and the young, which is much better than the control group without vaccination, especially after receiving multiple vaccinations. The incidence of AEs in the elderly is low, which proves that the vaccination in the elderly is safe and effective.

## Figures and Tables

**Table 1:** Characteristics of included studies.

Study	Vaccine	Participants (Young/Old)	Controls (Young/Old)	Age Range (Young/Old)	Country
J. Sadoff et al.	Ad26.COV2.S	162/161	82/81	18–55/≥65	Belgium and United States
Shengli Xia et al.	BBIBP-CorV	72/72	24/24	18–59/≥60	China
Jingxin Li et al.	BNT162b1	48/48	24/24	18–55/65–85	China
Maheshi N Ramasamy et al.	ChAdOx1	50/96	49/20	18–55/≥70	United Kingdom
Gang Zeng et al.	CoronaVac	430/256	110/47	18–59/≥60	China
Jiankai Liu et al.	KCONVAC	200/200	50/50	18–59/≥60	China
Neil Formica et al.	NVX-CoV2373	561/467	139/116	18–59/≥60	United States, Australia
Ya-Jun Shu et al.	V-01	360/360	80/80	18–59/≥60	China

## Data Availability

Additional data related to this paper may be requested from the authors.

## Supplementary Materials

The following supporting information can be downloaded at: https://www.mdpi.com/article/10.3390/vaccines11010033/s1, File S1: search strategy; File S2: PRISMA 2020 Checklist.
