# Supplementary material for: Efficacy and Safety of COVID-19 Vaccination in Older Adults: A Systematic Review and Meta-Analysis
Source: Vaccines (Basel). 2022 Dec 23;11(1):33. doi: 10.3390/vaccines11010033 (PMC9862835; doi:10.3390/vaccines11010033)
Supplement: Supplementary file 1 [file vaccines-11-00033-s001.zip › Supplementary File S1.pdf]

(COVID-19 Vaccines OR COVID 19 Vaccines OR Vaccines, COVID-19 OR COVID-19 Virus Vaccines OR COVID 19 Virus Vaccines OR Vaccines, COVID-19 Virus OR Virus Vaccines, COVID-19 OR COVID-19 Virus Vaccine OR COVID 19 Virus Vaccine OR Vaccine, COVID-19 Virus OR Virus Vaccine, COVID-19 OR COVID19 Virus Vaccines OR Vaccines, COVID19 Virus OR Virus Vaccines, COVID19 OR COVID19 Virus Vaccine OR Vaccine, COVID19 Virus OR Virus Vaccine, COVID19 OR COVID19 Vaccines OR Vaccines, COVID19 OR COVID19 Vaccine OR Vaccine, COVID19 OR SARS-CoV-2 Vaccines OR SARS CoV 2 Vaccines OR Vaccines, SARS-CoV-2 OR SARS-CoV-2 Vaccine OR SARS CoV 2 Vaccine OR Vaccine, SARS-CoV-2 OR SARS2 Vaccines OR Vaccines, SARS2 OR SARS2 Vaccine OR Vaccine, SARS2 OR Coronavirus Disease 2019 Vaccines OR Coronavirus Disease 2019 Vaccine OR Coronavirus Disease 2019 Virus Vaccine OR Coronavirus Disease 2019 Virus Vaccines OR Coronavirus Disease-19 Vaccines OR Coronavirus Disease 19 Vaccines OR Vaccines, Coronavirus Disease-19 OR Coronavirus Disease-19 Vaccine OR Coronavirus Disease 19 Vaccine OR Vaccine, Coronavirus Disease-19 OR COVID 19 Vaccine OR Vaccine, COVID 19 OR 2019-nCoV Vaccine OR 2019 nCoV Vaccine OR Vaccine, 2019-nCoV OR 2019 Novel Coronavirus Vaccines OR 2019 Novel Coronavirus Vaccine OR 2019-nCoV Vaccines OR 2019 nCoV Vaccines OR Vaccines, 2019-nCoV OR COVID-19 Vaccine OR Vaccine, COVID-19 OR SARS Coronavirus 2 Vaccines) AND (older OR elderly OR older adult OR aged) AND (random OR placebo OR double-blind)
